# International Network for Comparison of HIV Neutralization Assays: The NeutNet Report

**DOI:** 10.1371/journal.pone.0004505

**Published:** 2009-02-20

**Authors:** Eva Maria Fenyö, Alan Heath, Stefania Dispinseri, Harvey Holmes, Paolo Lusso, Susan Zolla-Pazner, Helen Donners, Leo Heyndrickx, Jose Alcami, Vera Bongertz, Christian Jassoy, Mauro Malnati, David Montefiori, Christiane Moog, Lynn Morris, Saladin Osmanov, Victoria Polonis, Quentin Sattentau, Hanneke Schuitemaker, Ruengpung Sutthent, Terri Wrin, Gabriella Scarlatti

**Affiliations:** 1 Unidad de Immunopatologia del SIDA, Instituto de Salud Carlos III, Madrid, Spain; 2 Laboratory of AIDS and Molecular Immunology, Fundação Oswaldo Crusz, Rio de Janeiro, Brazil; 3 Viral Evolution and Transmission Unit, San Raffaele Scientific Institute, Milan, Italy; 4 Department of Microbiology, Virology Unit, Institute of Tropical Medicine, Antwerp, Belgium; 5 Department of Microbiology, Dermatology and Infection, University of Lund, Lund, Sweden; 6 National Institute for Biological Standards and Control, Potters Bar, Hertfordshire, United Kingdom; 7 Institute of Virology, University of Leipzig, Leipzig, Germany; 8 Unit of Human Virology, San Raffaele Scientific Institute, Milan, Italy; 9 Department of Surgery, Duke University Medical Center, Durham, North Carolina, United States of America; 10 Pathogénie des infections persistantes, University Louis Pasteur, Strasbourg, France; 11 National Institute for Communicable Diseases, Johannesburg, South Africa; 12 WHO-UNAIDS HIV Vaccine Initiative (IVR/HVI), World Health Organization, Geneva, Switzerland; 13 Department of Vaccine Research, Henry Jackson Foundation for the Advancement of Military Medicine, Rockville, Maryland, United States of America; 14 Sir William Dunn School of Pathology, University of Oxford, Oxford, United Kingdom; 15 Department of Experimental Immunology, Sanquin Research, Landsteiner Laboratory, Center for Infectious Diseases and Immunity Amsterdam (CINIMA) at the Academic Medical Center of the University of Amsterdam, Amsterdam, the Netherlands; 16 Faculty of Microbiology, Siriraj Hospital, Mahidol University, National HIV Repository and Bioinformatic Center, Bangkok, Thailand; 17 Monogram Biosciences Inc, South San Francisco, California, United States of America; 18 New York University School of Medicine, New York, New York, United States of America; New York University School of Medicine, United States of America

## Abstract

**Background:**

Neutralizing antibody assessments play a central role in human immunodeficiency virus type-1 (HIV-1) vaccine development but it is unclear which assay, or combination of assays, will provide reliable measures of correlates of protection. To address this, an international collaboration (NeutNet) involving 18 independent participants was organized to compare different assays.

**Methods:**

Each laboratory evaluated four neutralizing reagents (TriMab, 447-52D, 4E10, sCD4) at a given range of concentrations against a panel of 11 viruses representing a wide range of genetic subtypes and phenotypes. A total of 16 different assays were compared. The assays utilized either uncloned virus produced in peripheral blood mononuclear cells (PBMCs) (virus infectivity assays, VI assays), or their Env-pseudotyped (gp160) derivatives produced in 293T cells (PSV assays) from molecular clones or uncloned virus. Target cells included PBMC and genetically-engineered cell lines in either a single- or multiple-cycle infection format. Infection was quantified by using a range of assay read-outs that included extracellular or intracellular p24 antigen detection, RNA quantification and luciferase and beta-galactosidase reporter gene expression.

**Findings:**

PSV assays were generally more sensitive than VI assays, but there were important differences according to the virus and inhibitor used. For example, for TriMab, the mean IC50 was always lower in PSV than in VI assays. However, with 4E10 or sCD4 some viruses were neutralized with a lower IC50 in VI assays than in the PSV assays. Inter-laboratory concordance was slightly better for PSV than for VI assays with some viruses, but for other viruses agreement between laboratories was limited and depended on both the virus and the neutralizing reagent.

**Conclusions:**

The NeutNet project demonstrated clear differences in assay sensitivity that were dependent on both the neutralizing reagent and the virus. No single assay was capable of detecting the entire spectrum of neutralizing activities. Since it is not known which *in vitro* assay correlates with *in vivo* protection, a range of neutralization assays is recommended for vaccine evaluation.

## Introduction

It is well established that neutralizing antibodies play a pivotal role in mediating protection against a range of virus infections including polio, measles, hepatitis and influenza [1] and it is a long held and widespread belief that they probably contribute to protection from human immunodeficiency virus type-1 (HIV-1) infection and/or disease [2]. Evidence in favor of a beneficial effect of HIV-1 neutralizing antibodies has been presented over the years [3], [4], [5], [6], [7], [8]. Despite this, early moves towards vaccine clinical studies in the early 1990s were discouraged by the limited titer and very narrow specificity of neutralizing antibodies induced by natural infection or immunization if neutralization was detected at all [9], [10], [11], [12]. Furthermore, the high level of genetic variability of the virus and its escape from the neutralizing antibody response are well documented and have further discouraged the HIV-1 vaccine field from considering the induction of humoral immunity as a pre-requisite for an effective HIV-1 vaccine [13], [14]. Consequently, in the late 1990s and the early years of this century vaccine efforts were mainly focused on eliciting a cellular immune response but, unfortunately, these have also failed to provide effective protection against HIV-1 [15], [16].

Over the years a wide range of HIV-1 neutralization assays and variants thereof have been developed and described in the literature. It became apparent by the early 1990s that HIV-1 neutralization assays and reagents should be compared and evaluated and this was best done by international networks [17], [18]. Analogously the World Health Organization (WHO) Network for HIV Isolation and Characterization undertook detailed genetic, biological and immunological characterization of globally prevalent and epidemiologically important HIV-1 isolates. These and other studies from several other laboratories led to the conclusion that antigenic variability may not present such an insurmountable obstacle to vaccine development, and since broadly cross-neutralizing antibodies can be detected in some HIV-1-infected individuals, these should be sought for in the context of HIV-1 vaccine development [19], [20], [21]. A WHO/UNAIDS consultation on regulation and clinical evaluation of HIV/AIDS preventive vaccines held in March 2001 recommended that a consensus be sought on methods to assess serological and cellular immune responses. This resulted in a WHO/UNAIDS workshop being convened on ‘Progress in the development and standardization of methods to measure HIV-1 neutralizing antibodies in HIV vaccine research and clinical trials’ at the San Raffaele Scientific Institute in Milan, Italy, in 2003, and was attended by 18 participants from 12 different countries from Europe, Africa, Asia and the Americas. The primary achievements of this meeting were to prepare recommendations on priorities for the standardization and quality control of HIV-1 neutralization assays and to organize an international multi-laboratory collaborative study to compare neutralization methods using a selected panel of international HIV-1 isolates and serologic reagents. Subsequently in 2004, a group of 11 laboratories, performing a range of different techniques to measure neutralizing antibodies, proceeded with the co-ordination of an international collaborative study, called NeutNet, aimed at the standardization of HIV-1 neutralization assays to be used in vaccine research and clinical trials. The group has been extended over the years to 15 laboratories and has completed the first phase of the study using different monoclonal antibodies (Mabs) and soluble (s)CD4 tested against 11 HIV-1 isolates and their clonal derivatives in 16 different assays.

As described in a recent minireview [22], efforts to characterize HIV-1 neutralization assays and reagents have been carried out by other consortia such as the studies initiated by the Laboratory Standardization Subcommittee for the Global HIV AIDS Vaccine Enterprise (GHAVE). Data were obtained essentially from two HIV-1 neutralization assays, one using primary HIV-1 isolates and peripheral blood mononuclear cells (PBMC assay) for infection and the other using pseudoviruses derived from corresponding isolates and the TZM-bl reporter cell line. The results showed that the degree of correlation between the two assays was dependent on the reagents used for neutralization. The NeutNet study, comparing a broader range of neutralization assays, has reached at a similar conclusion. A summary of these studies was presented at the global workshop on Standardization of HIV Neutralization Assays for Use in Vaccine Research and Clinical Trials, held at Varese, Italy, on March 17–18, 2007 [23]. The present article describes the results of the first phase of the NeutNet study and provides recommendations for the use of a range of neutralization assays including pseudovirus/recombinant virus, HIV-1 isolates, primary cells (PBMC, and macrophages) and cell lines, as well as new approaches to evaluate inhibitory antibodies (such as plaque reduction, cell-to-cell fusion assays, and complement inhibition assays).

## Methods

### Neutralization Assays

The methodologies used in this study are listed in Table 1 and differences between assay protocols in supplementary Table S1 and supplementary Figure S1. Detailed protocols are available at the EUROPRISE website www.europrise.org. The conventional PBMC based assay [24], [25], [26], [27] with readout based on p24 antigen production involves multiple rounds of virus replication, has a moderate reproducibility and sensitivity, is time-consuming and cumbersome to perform but involves the most physiological target cell. An alternative readout can be the measurement of viral RNA, which shortens the time by several days [28], [29]. Intracellular (IC) p24 antigen determination in infected PBMC cultures may be run as a single round assay with increased sensitivity, reproducibility and speed but it is not easy to perform [30]. The method of measuring ICp24 was also applied to other target cells, like macrophages [31]. Plaque reduction assays use either U87.CD4 or GHOST(3) cells engineered to express coreceptors for HIV [32], [33]. In U87.CD4 cells the syncytium-inducing capacity of HIV is exploited, while infected GHOST(3) cells turn green due to the activation of the GFP gene linked to the HIV-2 LTR. These assays are single round, highly reproducible, easy to perform, with sensitivity comparable to the PBMC assay, but require a shorter time. The fusion assay is based on fusion of effector cells expressing the native HIV-1 envelope on their surface (PM1 persistently infected with HIV-1) with target cells expressing the appropriate receptors (initially NIH-3T3 mouse fibroblasts or HeLa human epithelial cells stably expressing human CD4, CCR5 and/or CXCR4). The readout is measurement of ß-galactosidase activity [34]. Pseudovirus (PSV)-based assays exist in a number of variant assay formats using different target cells [35], [36], [37], [38]. A selected molecular clone is tested in a single round assay with luciferase readout that results in short-term assays with high reproducibility and sensitivity. Plasmid production and producer cell line culture history are crucial criteria and influences the results. Due to this a fairly large inter-laboratory variation has been documented [23]. Finally, assays using recombinant viruses have also been included [39], [40], [41]. This assay type was run with two different starting materials, *env* sequences were amplified either from culture supernatants or from cloned plasmid.

**Table 1:** Neutralization assays and their characteristics.

	Assay	Cell target	Infection	Ab persistence^3^	Read-out assay	Day^4^	Virus type	Lab^7^
**Virus Infectivity Assay**	Extra cellular p24	PBMC	MR	24 hr	ELISA	7	Isolate	3B, 5A
				constant		7, 10		7
				24 hr		14		6B
	Intra cellular p24		SR	constant	Flow Cytometry	2	High Titer Isolate	8
	Viral RNA		MR	constant	Real Time PCR	4	Isolate	11
	Plaque formation	Ghost/U87^1^	SR	24 hr	Microscopy: Green (Ghost) or Syncytial (U87) cells	3	Isolate	9
	Fusion	3T3.T4. CCR5/CXCR4	SR^2^	constant	β-Galactosidase	2 hr	Isolate	3A
**Pseudotyped Virus Based Assay**	Pseudotyped Virus	U87^1^	SR	constant	Luciferase	3	Env plasmid^5^	13, 4B
		TZMbl				2		2, 5B, 10
		3T3.T4. CCR5/CXCR4				2		1
		Ghost				3		6A
	Recombinant Virus	U87^1^	SR	constant	Luciferase	3	Isolate^6^	4A
		U87^1^	MR			5		12

MR = multiple round, SR = single round.

1 cells are stably transfected with CD4 and CCR5 or CXCR4.

2 Limited to cell-surface envelope/receptor interaction.

3 Time of incubation of the inhibitor/antibody with the virus and cells before washout.

4 Day at which read-out was performed; hr means hours when indicated.

5 Env expression plasmids (obtained through NIBSC).

6 Env was PCR amplified starting from culture supernatant.

7 Laboratory code.

### Inhibitory reagents

Mabs and soluble sCD4 were distributed by the Programme EVA Centre for AIDS Reagents (CFAR) NIBSC, UK. Mabs 2F5, 2G12 and 4E10 were kindly provided by Dr. D Katinger, Polymun Scientific GmbH, Austria, b12 by Dr D Burton, The Scripps Research Institute, USA and 447-52D by Dr S Zolla-Pazner, New York University Medical Center, USA. The sCD4 was purchased from Progenics Pharmaceuticals Inc, USA. Three monoclonal antibody reagents were tested including TriMab, an equal mixture of the three Mabs IgG1b12, 2G12 and 2F5; Mabs 447-52D and 4E10. The Mabs and sCD4 were tested at starting concentrations of 25 µg/ml and 10 µg/ml, respectively, followed by five 2-fold dilutions. Mab IgG1b12 is directed to the CD4-binding domain of HIV-1 envelope gp120 [42], 2G12 is directed to a 1→2mannose residues of gp120 [43], while 2F5 and 4E10 are specific for the transmembrane-proximal region of gp41 [44], [46]. Antibody 447-52D is specific for the third variable loop (V3) of gp120 [45]. The integrity of the Mabs was confirmed by PAGE gels and their HPLC profile established (data not shown).

### Viruses

**Figure 1:**
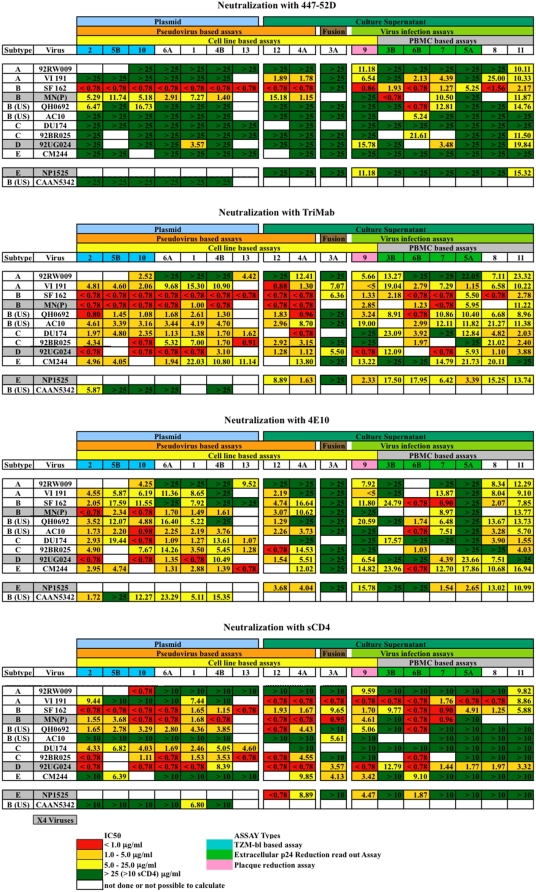
Mean inhibitory concentration (IC) 50 values for duplicate assays performed with virus and reagent as indicated. Each column represents the results obtained with one assay. The cells are color coded: green, poor or no neutralization IC50>25 µg/ml (>10 µg/ml for sCD4); yellow, IC50 5–25 µg/ml (5–10 µg/ml for CD4); orange, IC50 1–5 µg/ml; red, IC50<1 µg/ml. White cells occur where no results are available. Assays are grouped on the basis of several criteria: 1) the use of plasmids or culture supernatants as a source for HIV-1; 2) fusion based assays or infection based assays, either with pseudotyped virus or replication competent virus; and 3) the use of cell lines or PBMC. Laboratories performing the assays are numbered (see Table 1 for reference) and color coded; blue: TZM-bl assays or PSV/plasmid assays; green: PBMC assays using extracellular p24 as readout; pink: plaque reduction assay. In the listing of viruses to the left, the cells of X4 viruses are labeled grey, the cells of R5 viruses are white. NP1525 was available only as virus supernatant whereas, CAAN5342 as clonal derivative.

Twelve HIV-1 isolates and/or their clonal derivatives were used (see Figure 1 for listing). The viruses chosen represented different HIV-1 subtypes, varying neutralization sensitivity and coreceptor usage and included several presently used as vaccine strains. Three of the subtype B viruses, B (US), are from a virus panel currently in use for evaluating vaccine candidates [47]. Viruses were prepared and supplied to each participant by CFAR thereby ensuring that all the laboratories had a common starting material. Each participant laboratory subsequently expanded the stock/plasmid as needed and titered for use. NP1525 was available only as virus supernatant and CAAN5342 was only available as a clonal derivative. Each laboratory received a panel of 11 different viruses for testing. Virus isolates 92RW009, 92BR025 and 92UG024 originated from the WHO/UNAIDS HIV Network and were provided to CFAR by the NIH AIDS Research and Reference Reagent Program (ARRRP).

### Statistical analysis

Analysis was based on the raw assay data returned by participating laboratories. Each laboratory was requested to perform twice the assays according to their standard protocol, with all dilutions tested at least in duplicates. The 50%, 75% and 90% inhibitory concentrations (IC50, IC75 and IC90) were calculated with a linear interpolation method, using the mean of the duplicate responses.

The linear interpolation method was implemented in an Excel spreadsheet, allowing consistent calculation of the IC50 values that was across laboratories. The assay readout equivalent to the IC50 was calculated as half the assay readout with no antibody present (similarly for IC75 ad IC90). The dilution interval containing the IC50 was identified, with assay readout for adjacent dilutions being above and below the 50% readout. The assay readouts for the dilutions above and below the IC50 were joined with a straight line, plotted against the log concentration of antibody. The position where the line crossed the 50% assay readout was taken as the estimate of IC50. Where the IC value was outside the range of concentrations tested, it was recorded as either greater than the highest concentration used, or less than the lowest concentration, as appropriate. Where the assay data were variable, and the observed dose-response crossed the relevant percentage inhibition level (e.g. 50% inhibition for IC50) more than once, no IC value was calculated. Absence of a calculated IC value may therefore be due to a laboratory not testing a particular combination of virus and antibody, or to the resulting assay data being too variable to allow a calculation. The variable data quality precluded the use of more sophisticated curve-fitting models for calculation of IC values.

For each laboratory, a geometric mean IC value for the repeat tests was calculated. For each virus and antibody combination, an overall geometric mean of the individual laboratory means was calculated, along with the minimum, maximum, and range between laboratories. To allow calculations of the geometric means, any IC value that was greater than the highest concentration used were taken as equal to the next two-fold dilution step, so results recorded as >25 were taken as equal to 50. Similarly, IC values that were below the lowest concentration were taken as the next two-fold dilution step. IC values that were below 0.78 are recorded as <0.78, even if a lower IC value could be calculated from an extended dilution series. Although this may not fully reflect the differences in sensitivities of different assays, it does allow a consistent comparison between different laboratories and groups of assay methods, without introducing any bias from the range of concentrations included in the assays.

## Results

### Calculated Inhibitory Concentration values

The majority of assays had IC90 values above the highest concentration of inhibitor used. The IC75s were also above the highest concentration tested for many assays, particularly for 447-52D and sCD4. The calculated IC50s were used for all subsequent analyses.

### Overall neutralization assay results

The overall pattern of neutralization of the different viruses by the four reagents is shown in Figure 1, as a color-coded table. The table values are the geometric mean IC50 (µg/ml) from the indicated laboratory for a given inhibitor tested with each virus. The cells range from green representing weak or no neutralization (IC50>25 µg/ml; >10 µg/ml for sCD4) to red representing strong neutralization (IC50<1.0 µg/ml). Empty (white) cells indicate a data point not reported by a participant, or a place where data points were too variable to allow the calculation of an IC50 value. The laboratories are grouped according to the type of assay that they performed, indicated by titles at the top of the tables.

TriMab, a mixture of three Mabs, was the most effective and broadly neutralizing of the four reagents used. Given that Mab 447-52D is specific for viruses that carry the GPGR motif at the tip of the V3 loop [2], a characteristic of most subtype B viruses but of few non-B viruses, it displayed the most consistent reactivity with two of four subtype B viruses and VI191 (subtype A with a GPGR V3 motif), but little activity with viruses of other subtypes.

As illustrated in Figure 1 different viruses showed a differential sensitivity to neutralization by the various Mabs and sCD4. For example, SF162 was generally easier to neutralize than other viruses, showing strong neutralization by 447-52D, but much less so by 4E10. Moreover, there were significant differences in the ability of the different assays to detect neutralization. For example, the fusion assay of laboratory 3A had a strikingly higher sensitivity threshold than the other assays when testing Mabs, but very comparable sensitivity when testing sCD4.

### Intra-laboratory variation

**Figure 2:**
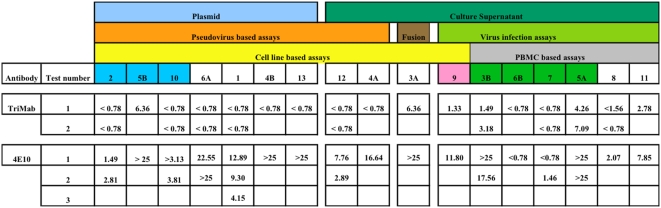
Intra-lab variation of neutralization performed with SF162. Light blue coded laboratory performed TZM-bl assay; green coded laboratory performed extracellular p24 reduction readout assay and pink coded laboratory performed placque reduction assay. Assays are grouped as in Figure 1.

Figure 2 shows as an example the calculated IC50s from individual tests performed by laboratories for SF162 with TriMab and 4E10. Many laboratories only provided results from a single assay, and a full assessment of intra-laboratory repeatability is not possible. For laboratories that did return data from repeat assays, the IC50s were generally within a 2–4 fold range. This is much lower than the variation between laboratories, even between laboratories performing nominally the same assay. However, given the limited nature of the data it is not possible to draw any firm conclusions.

### Inter-laboratory and inter-assay variation

The aim of the present study was to compare the performance of a wide variety of HIV-1 neutralization assays as performed in different laboratories (Table 1). The viruses and inhibitory reagents were provided by a common source and there were certain standards requested in the assay setup including the starting of the titrations at 25 µg/ml for the Mabs and 10 µg/ml for sCD4, with subsequent 2-fold dilution series. Inter-laboratory comparisons were made on a range of dilutions common to all laboratories. IC50s below 0.78 are indicated as <0.78.

Direct comparison of the PSV and VI assays was possible with 10 viruses. The results of the fusion assay were not included in these calculations. The PSV assays were generally more sensitive than VI assays, but there were important differences according to the virus as well as the neutralizing reagent.

Tables 2, 3 and 4 list the overall mean IC50s for neutralization by TriMab, along with the fold-range (max/min) between laboratories. Since for many of the laboratories the mean IC50s were outside the range of concentrations used, the quoted fold-range is a minimum observed range (for example, a range from 2.5 to >25 is presented as >10-fold; the true range is unknown). For TriMab, the mean IC50 was lower for PSV than for VI assays in all cases (Table 2). However, neutralization of some viruses with 4E10 or sCD4 suggested greater sensitivity for those reactions of the VI compared to the PSV assay (e.g., SF162 *vs.* 4E10 lower, *vs.* sCD4 similar; whereas VI191 *vs.* 4E10 similar and *vs.* sCD4 lower IC50s in VI assays than in PSV assays; Figure 1). For TriMab, with viruses for which a comparison of ranges between laboratories could be made (QH0692 and AC10), the PSV assays performed in different laboratories were in better agreement with each other than the VI assays, whereas for VI191 agreement between laboratories was equally poor. For CM244 the opposite was true since the VI assays were in better agreement than the PSV assays (Table 2). For other viruses, no comparisons could be made since several laboratories had IC50 values outside the range of concentrations (<0.78 or >25). This was also true for 4E10 and sCD4.

**Table 2:** Inter-Laboratory comparisons with TriMab^a^.

Pseudovirus Based Assays (PSV)^c^						Virus Infection Assays (VI)^d^				
Virus	N lab^b^	Mean IC50	Min IC50	Max IC50	Fold Range	N lab^b^	Mean IC50	Min IC50	Max IC50	Fold Range
**92RW009**	7	14.98	2.52	>25	>9.9	7	16.52	5.66	>25	>4.4
**VI 191**	8	4.13	0.88	15.30	17.5	7	5.22	1.15	19.04	16.6
**SF 162**	9	<0.78	<0.78	<0.78	-	7	1.33	<0.78	5.50	>7.1
**MN(P)**	7	<0.78	<0.78	1.00	>1.3	5	2.80	<0.78	11.22	>14.4
**QH0692**	8	1.37	0.80	2.61	3.3	7	5.23	<0.78	10.86	>13.9
**AC10**	8	4.14	2.96	8.70	2.9	6	11.19	2.99	21.27	7.1
**DU174**	8	1.66	<0.78	4.80	>6.2	7	10.19	2.03	>25	>12.3
**92BR025**	8	2.52	<0.78	7.00	>9.0	5	10.40	1.97	>25	>12.7
**92UG024**	7	0.88	<0.78	3.10	>4.0	6	2.22	<0.78	12.09	>15.5
**CM244**	7	7.57	1.94	22.03	11.3	7	23.24	13.22	>25	>1.9

a all values of the ICs are expressed as µg/ml. A dash indicates: not possible to calculate.

b Number of laboratories performing the assay.

c Laboratories involved: 1, 2, 4A, 4B, 5B, 6A, 10, 12, 13.

d Laboratories involved: 3B, 5A, 6B, 7, 8, 9, 11.

**Table 3:** Inter-Laboratory comparisons with TriMab^a^.

Culture Supernatant Based Assays^c^						Plasmid Based Assays^d^				
Virus	N lab^b^	Mean IC50	Min IC50	Max IC50	Fold Range	N lab^b^	Mean IC50	Min IC50	Max IC50	Fold Range
**92RW009**	9	17.40	5.66	>25	>4.4	5	13.12	2.52	>25	>9.9
**VI 191**	9	3.67	0.88	19.04	21.7	6	6.48	2.06	15.30	7.4
**SF 162**	9	1.10	<0.78	5.50	>7.1	7	<0.78	<0.78	<0.78	-
**MN(P)**	7	1.82	<0.78	11.22	>14.4	5	<0.78	<0.78	1.00	>1.3
**QH0692**	9	3.86	<0.78	10.86	>13.9	6	1.39	0.80	2.61	3.3
**AC10**	8	9.18	2.96	21.27	7.2	6	3.87	3.16	4.70	1.5
**DU174**	8	7.15	<0.78	>25	>32	7	1.92	1.13	4.80	4.3
**92BR025**	7	7.31	1.97	>25	>12.7	6	2.37	<0.78	7.00	>9.0
**92UG024**	8	1.90	<0.78	12.09	>15.5	5	0.78	<0.78	3.10	>4.0
**CM244**	8	21.78	13.22	>25	>1.9	6	6.85	1.94	22.03	11.3

a all values of the ICs are expressed as µg/ml. A dash indicates: not possible to calculate.

b Number of laboratories performing the assay.

c Laboratories involved: 3B, 4A, 5A, 6B, 7, 8, 9, 11, 12.

d Laboratories involved: 1, 2, 4B, 5B, 6A, 10, 13.

**Table 4:** Inter-Laboratory comparisons with TriMab^a^.

TZM-bl Assays^c^						Other Plasmid Based Assays^d^				
Virus	N lab^b^	Mean IC50	Min IC50	Max IC50	Fold Range	N lab^b^	Mean IC50	Min IC50	Max IC50	Fold Range
**92RW009**	1	2.52	2.52	2.52	-	4	19.82	4.42	>25	>5.7
**VI 191**	3	3.57	2.06	4.81	2.3	3	11.73	9.68	15.3	1.6
**SF 162**	3	<0.78	<0.78	<0.78	-	4	<0.78	<0.78	<0.78	-
**MN(P)**	3	<0.78	<0.78	<0.78	-	2	<0.78	<0.78	1.00	>1.3
**QH0692**	3	1.08	0.80	1.45	1.8	3	1.79	1.30	2.61	2.0
**AC10**	3	3.67	3.16	4.61	1.5	3	4.08	3.44	4.70	1.4
**DU174**	3	2.81	1.97	4.80	2.4	4	1.44	1.13	1.70	1.5
**92BR025**	2	1.75	<0.78	4.34	>5.6	4	2.76	0.91	7.00	7.7
**92UG024**	2	<0.78	<0.78	<0.78	-	3	0.98	<0.78	3.10	>4.0
**CM244**	2	4.48	4.05	4.96	1.2	4	8.47	1.94	22.03	11.3

a all values of the ICs are expressed as μg/ml. A dash indicates: not possible to calculate.

b Number of laboratories performing the assay.

c Laboratories involved: 2, 5B, 10.

d Laboratories involved: 1, 4B, 6A, 13.

When comparing plasmid-based assays with culture supernatant-based assays (see Figure 1 for subdivision of assay types) the overall pattern was similar to the PSV *vs.* VI assay comparison (Table 3). Nevertheless, it was possible to compare ranges between laboratories for more viruses, and the plasmid-based assays were in better agreement with each other than the culture supernatant-based assays for VI191 and DU174 as well as for QH0692 and AC10. The mean IC50s with TriMab were lower in the plasmid-based assays than in culture supernatant-based assays for all but one virus (VI191), although the consistency of assays between laboratories was in favor of the plasmid-based assays (7-fold and 21.7-fold range, respectively, Table 3).

### Variation within plasmid based assays

The TZM-bl assay (performed by three laboratories, numbered 2, 5B and 10) was compared to all other plasmid-based assays (comprising four laboratories, numbered 6A, 1, 4B and 13). The results largely depended on the virus used (Figure 1 and Table 4). For TriMab, the TZM-bl assays gave good neutralization for all viruses, with good agreement between laboratories (fold range 1.2–2.4; with the exception of 92BR025 >5.6). For the other plasmid assays, the results depended on the virus, with poor neutralization for 92RW009, VI191 and CM244. For the other viruses, the results were similar to those of the TZM-bl assays. Likewise, 4E10 neutralized all viruses in the TZM-bl assays, but was weak or did not neutralize 92RW009, VI191 and SF162 in the other plasmid-based assays. For sCD4, VI191, AC10, CM244 and CAAN5342 showed poor or no neutralization with both sets of assays. For the other viruses and reagents, the overall neutralization profile was similar in the two types of assays (Figure 1).

### Variation within PBMC based assays

PBMC-based assays with different readouts were compared. Four laboratories used extracellular p24 (EC-p24) detection (laboratories 3B, 6B, 7 and 5A), while intracellular p24 (IC-p24) or RNA viral load by real-time PCR comprised the other group (laboratories 8 and 11). No general trend between assay types was evident, since the observed range was affected by the cut-off of 25 (or 10) µg/ml as the highest concentration of reagent tested.

There are several variables in the preparation of PBMC and in neutralization assay protocols (Table 1 and supplementary Table S1), which may have an impact on assay sensitivity. According to their sensitivity to neutralization of SF162 with TriMab or 4E10 (Tables 2, 3 and 4) laboratories could be divided into three categories. Sensitive, IC50 reached with <0.78 µg/ml Mab; resistant, high concentrations needed (TriMab) or highest concentration not sufficient (4E10) to reach IC50; and moderately sensitive, with IC50 values in between (sensitive<moderately sensitive<resistant). Of all the variables, the length of time during which the antibody was present correlated best with the sensitivity of the assay. The longer the antibody was present in the culture, the better the neutralization that was achieved. The three laboratories (numbered 6B, 7 and 8) that neutralized SF162 to the highest level had in common the constant presence of antibody or a 24-hour incubation period with antibody and virus prior to addition of the mixture to cells. With other viruses, however, this hierarchy was not maintained.

### Detailed comparisons within individual laboratory methods

#### Intracellular - p24 assay

The standard IC-p24 assay is based on PBMC infection, but it can also be adapted to measure neutralization using other target cells such as immature dendritic cells (DC) and monocyte-derived macrophages (MDM). Indeed, neutralizing Mabs had lower ICs on DC and even still lower ICs on MDM (CD4-T-cells<DC<MDM) [31]. For the three viruses VI191, SF162 and 92BR025 for which HIV-1 replication in MDM was sufficient, the Fc-gamma mediated inhibitory assay was performed. We found a 7.5 to more then 300% increase of IC50 or IC90 for the Mabs when macrophages were used as target cells instead of PBMC whereas similar IC50s and IC90s were recorded for sCD4 in the two cell systems (Table 5). These results support previous data obtained with neutralizing Mabs and sCD4 on the three other primary isolates BaL, Bx08 and TV1 [31].

**Table 5:** Comparisons of Intracellular p24 assay performed with CD4+ T cells and macrophages.

Virus	Cells	Neutralization (IC90; µg/ml)			
		477-52D	TriMAb	4E10	sCD4
**VI191**	**CD4 T Cells**	>25	>25	>25	1
	**Macrophages** 1	0.13±0.04	0.13±0.02	0.07±0.02	1±0
**SF162**	**CD4 T Cells**	6	2	>25	5
	**Macrophages**	0.1±0.1	0.03±0.03	0.08±0.04	5±1
**92BR025**	**CD4 T Cells**	>50	>25	>25	>25
	**Macrophages**	>25	10	0.8±0.4	28±4

1 Experiments were carried out on macrophages generated from 3 different donors. Results are the mean+/−standard deviation of two independent experiments.

#### Plaque reduction assays on GHOST(3) and U87.CD4 cells

For IC50 comparisons with the other assays the GHOST(3) cells expressing CCR5 or CXCR4 were used. In general, results of the plaque reduction assay fit in the middle range of IC50s of the other VI assays; better for some neutralizing reagents and viruses, worse for other neutralizing reagents and viruses (laboratory 9 in Figure 1 and Figure 2). This is in agreement with the observation that the neutralization sensitivity of assay types depended on both the virus and neutralizing reagent and, also with the plaque reduction assay, no general trend could be seen apart from this.

**Figure 3:**
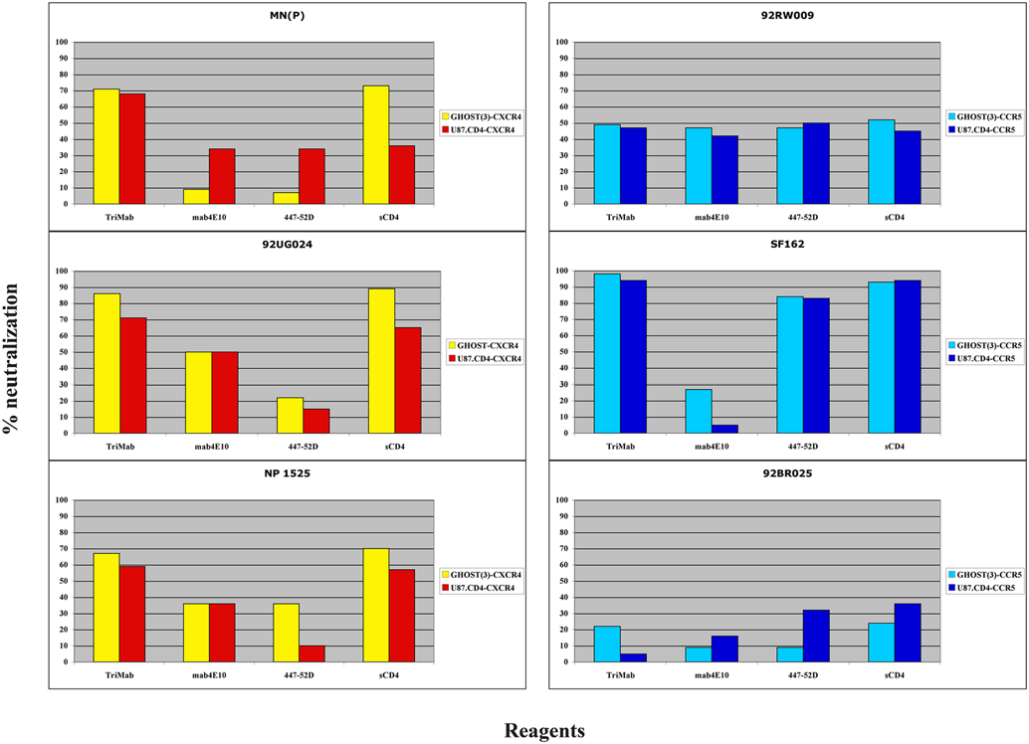
Percentage neutralization with Mabs and sCD4 performed in plaque reduction assay using GHOST(3) or U87.CD4 (CCR5- or CXCR4-expressing) cells of viruses as indicated. Cut-off is set at 30% neutralization (3 SD above the negative control, based on intra-assay variation). GHOST(3) cells contain GFP which is activated upon HIV infection and green cells can be counted in a fluorescence microscope 3 days after infection. The readout in HIV-infected U87.CD4 cultures is light microscopic counting of syncytial cells (single or groups of syncytia) after fixation and heamatoxylin staining.

We also compared the two cell lines, GHOST(3) and U87.CD4, for assaying HIV-1 neutralization sensitivity. This comparison was done at one single reagent concentration (5 µg/ml) and showed a close agreement of the results on the two cell types (Figure 3).

#### Fusion assay

The fusion assay almost invariably showed the highest threshold for the detection of neutralization, except for sCD4. Strikingly, when mouse 3T3 cells were used as target cells the anti-gp41 Mab 4E10 did not neutralize any of the viruses (laboratory 3A in Figure 1). Since few viruses were neutralized with TriMab as well, we considered the possibility that the anti-gp41 component of TriMab (Mab 2F5) could prevent neutralization by the other components.

To investigate whether the nature of the target cell was affecting neutralization, experiments were repeated with the human epithelial cell line (HeLa) endogenously expressing CXCR4 and engineered to express human CD4 and CCR5. The individual components of TriMab were tested separately to see whether anti-gp41 MAbs inhibited neutralization. The results of parallel experiments carried out with two viruses, one R5 and one X4 (QH0692 and 92UG024, respectively), showed that the sensitivity of the fusion assay to detect neutralization was greatly increased when using HeLa cells as target cells (Table 6). While QH0692 could not be neutralized in 3T3 cells with 25 µg/ml of TriMab, it was neutralized in HeLa cells with a similar IC50 as in all other assays. Mab 4E10 neutralized with IC50s comparable to other VI assays. Interestingly, 2F5, the other Mab directed to gp41, neutralized as well, as did the other components (2G12 and IgGb12) of TriMab tested individually. Neutralization of 92UG024 was enhanced on HeLa cells as compared to 3T3 cells with both TriMab and 4E10. We conclude that the anti-gp41 component of TriMab does not interfere with neutralization in the fusion assay and that the poor neutralization achieved in initial experiments was primarily due to the target cell type used in the assay.

**Table 6:** Effect of target cell on neutralization in the fusion assay.

Virus	Target cells^a^	Neutralization (IC50; µg/ml)				
		TriMab	4E10	2F5	2G12	IgG1 b12
**QH0692**	**3T3**	>25	>25	>25	>25	>25
	**HeLa**	4.03	13.38	13.26	6.12	7.18
**92UG024**	**3T3**	4.31	24.63	>25	3.86	>25
	**HeLa**	1.44	1.35	3.59	1.25	>25

a 3T3, mouse cell line engineered to express CD4 and CCR5 and CXCR4 [51].

HeLa, human cell line engineered to express CD4 and CCR5 [52].

#### Culture supernatants vs. plasmids in a recombinant virus assay

An assay based on recombinant viruses produced by co-infection of HEK-293 cells with a cloned *env* gp160 gene and an HIV-1 *env* vector expressing luciferase allowed the comparison of plasmid and culture supernatant derived virus populations (laboratory 4A and and 4B, respectively in Figure 1). All the DNA-derived and virus-derived *env* recombinant viruses gave similar levels of neutralization except for two viruses. The virus-derived recombinant of 92UG024 was 7-fold more sensitive to sCD4 than the plasmid derived one. For VI191 the virus-derived recombinant was between 7- and 74-times more sensitive to neutralization then the plasmid-derived recombinant by two of the Mabs and sCD4. Interestingly, the envelope sequences of the two *env*-variants, the virus- and plasmid-derived recombinant, of VI191 revealed significant differences at several residues, most notably in the V3 region. These differences likely contribute to the difference in sensitivity to neutralization. The envelope sequences from the other plasmid or culture supernatant derived viruses were compared and all clustered closely (data not shown).

## Discussion

The primary aim of this study was to co-ordinate activities aimed at comparing methods for the measurement of neutralizing antibodies to HIV-1 for use in HIV-1 research as well as in human clinical trials of candidate HIV/AIDS vaccines. The comparisons led us to the conclusion that at present no single assay can be recommended for the measurement of HIV-1 neutralization because the assay results vary significantly depending on both the virus and the reagent used. Different assays vary in their sensitivities for measuring of neutralization with the different virus-reagent combinations. Both the virus and the reagent used for neutralization influence the assay sensitivity. Since it is not known at present which *in vitro* assay best correlates with biological (*in vivo*) protection, a panel of neutralization assays has to be recommended for vaccine evaluation.

In the present study IC50 values were used for the comparisons. We also considered the use of IC75 or even IC90, but many of the assays would have been excluded from the comparison as 90% or even 75% neutralization was often not achieved in most of the assays used. In conventional VI assays with polyclonal reagents, such as patient sera or plasma, IC90 values are the parameters usually considered for measuring neutralization as this increased stringency parallels increased specificity. In the present study, however, we used defined reagents with known specificities, which allows comparisons at the level of IC50s. It is important to emphasize that in future comparisons of the assays with polyclonal reagents it may be mandatory to compare higher ICs during evaluation.

Although with some combinations of reagents PSV assays appeared to be more sensitive for the detection of neutralizing activity than VI assays, the use of molecularly-cloned pseudoviruses has the caveat of testing single clones and not a complex viral quasispecies as in the case of uncloned viral isolates. Single clones may give different results and may not be representative of the prevalent viruses in the quasispecies present in the corresponding isolate, and even less of the virus population circulating *in vivo* in the infected individual. This problem might be overcome, at least in part, by using a pool of amplified clones rather than single clones, as occurred in the tests performed by partner 4 and 12. One may also consider that the envelope spike density and stability of pseudoviruses is different from those of primary isolates and this may account for a higher sensitivity to neutralization. Continued careful comparison of the assays is therefore very important.

The use of PBMC as target cells in neutralization assays is the closest *in vitro* approximation to the *in vivo* situation. However, assays using PBMC from different donors isolated on different days show great variability in sensitivity as clearly reflected by our inter-laboratory comparisons. In addition, the number of donors included in each test, the time of incubation with the virus and the readout of the assay were all major variables amongst the different laboratories. Of all the variables, the length of time during which the inhibitor was present correlated best with the sensitivity of the assay. Again, however, this relationship seemed to be true for one virus (SF162), while other viruses did not follow this pattern. These data reinforce the conviction that there is a great need to standardize the PBMC assays for HIV-1 neutralization, an activity that is currently in progress in our network and other groups worldwide.

An encouraging result emerging from our study was that the plaque reduction assays gave similar results to the PBMC-based assays. Using cell lines engineered to express the HIV-1 co-receptors may provide useful model systems for HIV-1 neutralization. PBMC-produced cytokines and chemokines may add to the existing variables in a virus-dependent manner, relating to the sensitivity or resistance of the virus to inhibition by cytokines and/or chemokines [48]. The use of U87.CD4 or GHOST(3) cells excludes this variable and provides simple, reliable and cheaper assay systems. We are currently working on the conversion of the readout of the plaque reduction assays to an automatic and high-throughput readout.

Another aspect related to the nature of the target cells used in HIV-1-neutralization assays has emerged in connection with the fusion assay. We found that mouse 3T3 cells supported neutralization by sCD4 but not by Mabs. However, when we employed human HeLa cells as targets the Mabs acted with similar efficiency as in other assays. Of note, efficient neutralization by Mabs was also observed using another human cell line of B-lymphoid origin, 721–22 (not shown), suggesting that the sensitivity to Mab-mediated neutralization in the fusion assay depends on the specific characteristics of the cell lines used as targets, with human cells showing a greater sensitivity than murine cells. In contrast to the results obtained in the fusion assay with murine target cells, Mabs showed an increased inhibitory activity when macrophages were used as target cells instead of PBMC in VI assays. The macrophage-based assay detected neutralization mediated by the Fab as well as the Fcγ fragment of the immunoglobulin. Conceivably, the antibodies induced endocytosis and degradation of immune complexes via binding to FcγR1 receptor (CD64) on macrophages [49]. Moreover, CD64 engagement by immunoglobulins has been reported to trigger a negative regulatory signal that suppresses HIV-1 replication in macrophages. Whether these additional mechanisms contribute to HIV-1 inhibition in macrophages *in vivo* need to be investigated. It has also been described that the level of CCR5 but not CD4 expression on the cell surface determines neutralization by certain MAbs. For example, neutralization by 4E10 was less effective in HeLa cells engineered to express CCR5 at high levels as compared to low levels (comparable to those on CD4+ T lymphocytes) [50]. This question is particularly pertinent since in VI assays we used both cell lines and PBMC. However, such a relationship could not be established when comparing the plaque-reduction assays, performed in U87.CD4-CCR5 or GHOST(3)-CCR5 cells, with assays based on PBMC. In general, IC50s obtained in the plaque-reduction assays fitted in the middle of the spectrum of sensitivity of the other VI assays. Interestingly, IC50s obtained with 4E10 against 92RW009 or VI191 were at the lower end of the scale when using the cell line-based plaque reduction assay (Figure 1).

The present results provided the necessary starting platform for designing the future strategies of the NeutNet programme. In the next step, we plan to assay plasma, sera and purified IgG from human and animal sources in order to make comparison of the assays more complete. A reduced virus/clone panel of 8 isolates will be proposed, and a number of polyclonal human plasma preparations will be evaluated using different assays optimized according to the data so far obtained. Taken together, the comparison of 16 different HIV-1 neutralization assays within the framework of an international network, NeutNet, comprised of 15 laboratories, led us to the conclusion that at present no single assay can be recommended for use alone as a potential correlate of vaccine efficacy. Both the virus and the reagent used for neutralization (here monoclonal antibodies and sCD4) contribute to the outcome of assay sensitivity, which in certain combinations is also influenced by the target cell used. Until we gather more precise information on which assays may correlate with protective immunity, a panel of neutralization assays must be recommended for vaccine evaluation.

## Supporting Information

Figure S1 Characteristics of the pseudotyped virus based assays. Assays are grouped as in Figure 1. Plasmid backbone are all pNL4-3 derived. 1 Five two-fold serial dilutions were used in the neutralization assay.
(2.51 MB TIF)

Table S1 Characteristics of the PBMC based assays Footnote to Supplementary Table S1: 1 Stimulated prior freezing 2 number of donors used 3number of cells/ml of medium 4 only in medium of the neutralization assay 5 incubation time (hr) virus and inhibitory reagent of the neutralization assay 6 absorption time (hr) virus/inhibitory reagent mix on the cells before wash step 7 3 washes in case patient's sera/plasma are used 8 incubation time before virus infectivity is measured 9 culture period is 2 days for real-time PCR and 8 days for p24 antigen
(0.02 MB XLS)
