# Supplementary figures and images for: International Network for Comparison of HIV Neutralization Assays: The NeutNet Report
Source: PLoS One. 2009 Feb 20;4(2):e4505. doi: 10.1371/journal.pone.0004505 (PMC2640999; doi:10.1371/journal.pone.0004505)

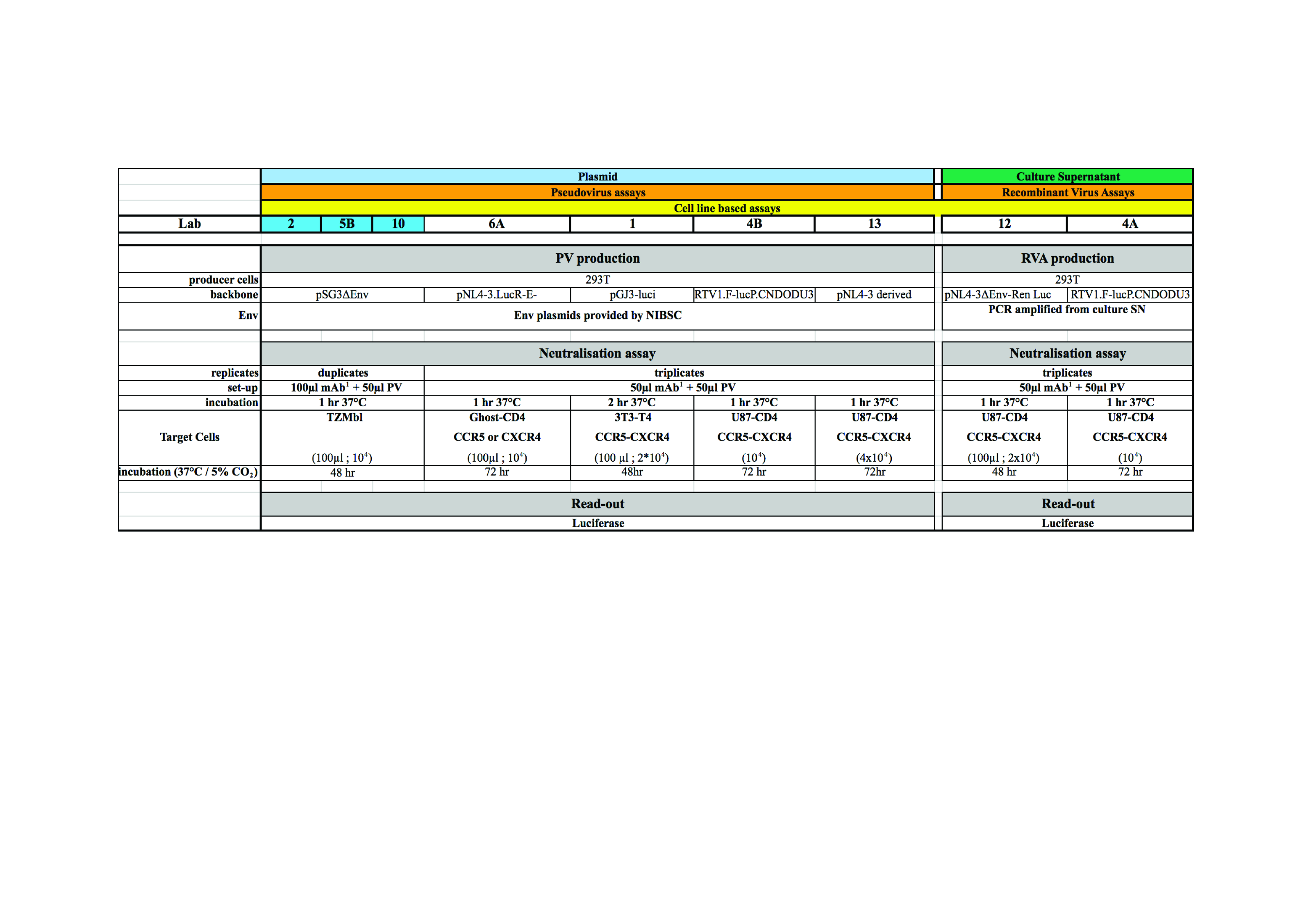

Supplement: Figure S1 — Characteristics of the pseudotyped virus based assays. Assays are grouped as in Figure 1. Plasmid backbone are all pNL4-3 derived. 1 Five two-fold serial dilutions were used in the neutralization assay. (2.51 MB TIF) [file pone.0004505.s001.tif]
